# Does Allergy Break Bones? Osteoporosis and Its Connection to Allergy

**DOI:** 10.3390/ijms21030712

**Published:** 2020-01-21

**Authors:** Maria Maddalena Sirufo, Mariano Suppa, Lia Ginaldi, Massimo De Martinis

**Affiliations:** 1 Department of Life, Health and Environmental Sciences, University of L’Aquila, 67100 L’Aquila, Italy; maddalena.sirufo@gmail.com (M.M.S.); lia.ginaldi@cc.univaq.it (L.G.); 2 Allergy and Clinical Immunology Unit, Center for the diagnosis and treatment of Osteoporosis, AUSL 04 Teramo, 64100 Teramo, Italy; 3 Department of Dermatology, Hôpital Erasme, Université Libre de Bruxelles, 1070 Brussels, Belgium; dr.marianosuppa@gmail.com

**Keywords:** allergy, osteoporosis, bone, hypersensitivity, IgE, mastocyte, eosinophil, eczema, asthma, urticaria, skeletal health, translational immunology

## Abstract

Osteoporosis and allergic diseases are important causes of morbidity, and traditionally their coexistence has been attributed to causality, to independent processes, and they were considered unrelated. However, the increasing knowledge in the field of osteoimmunology and an increasing number of epidemiological and biological studies have provided support to a correlation between bone and allergy that share pathways, cells, cytokines and mediators. If the link between allergic pathology and bone alterations appears more subtle, there are conditions such as mastocytosis and hypereosinophilic or hyper-IgE syndromes characterized by the proliferation of cells or hyper-production of molecules that play a key role in allergies, in which this link is at least clinically more evident, and the diseases are accompanied by frank skeletal involvement, offering multiple speculation cues. The pathophysiological connection of allergy and osteoporosis is currently an intriguing area of research. The aim of this review is to summarize and bring together the current knowledge and pursue an opportunity to stimulate further investigation.

## 1. Introduction

Osteoporosis and allergies are diseases with a high prevalence in the general population and they represent both a major problem of public health [1,2,3,4]. A number of comorbidities are associated with fracture risk, including eczema, pollen-allergy, asthma, and chronic respiratory diseases [5,6,7]. Initially, long-term steroid therapy [8] was the only clinical correlation between the diseases, but recently several other relevant relationships have been described. Furthermore, the effects of leukotriene modifiers and antihistamine on bone formation and resorption represent another connection between bone metabolism and atopy. The possible negative actions of therapies commonly used is not the only relationship between allergy and bone. Available data confirm a strict connection between bone and allergy, and we suggest paying additional attention to bone loss in allergic patients. Currently, research in this field is growing and the results are very encouraging and fruitful: studies on allergy and osteoporosis are becoming very intriguing. Osteoporosis treatment, once the diagnosis is made, hardly reverts to the baseline patient’s bone density, thus prevention and early treatment after identification of primary risk factors is the best therapeutic approach. We discuss and try to shed light on the possible interactions of immune and bone systems in the allergic-mediated diseases. The close interaction of the two systems have the consequence that the disease of one affects the other. A variety of functions and molecules, such as transcription factors, chemokines, cytokines, and signaling molecules, are shared by bone and immune systems. Immune and bone cells reciprocally regulate each other and hematopoiesis.

## 2. Osteoporosis and Allergy

Osteoporosis is a disease with great impact, whose incidence is greatly increasing in recent decades. A disorder of bone tissue microarchitecture together with loss of bone mass characterize osteoporosis, a chronic systemic disease of the skeleton, which consequently leads to fracture tendency due to increased bone fragility. Epidemiological data show that it affects approximately 200 million people in the world, the risk is higher in women: 30% compared to 12% of man suffering of osteoporosis at any age (one woman every three and one man every eight over the age of fifty). In postmenopausal women older than 60, the prevalence is around 40–50%. In Europe (EU 27), the prevalence is around 22 million women and 5.5 million men aged 50–84 years. In the United States, osteoporosis affects one woman every four and more than one man every eight over the age of fifty [9]. The estimated number of osteoporotic hip fractures worldwide by the World Health Organization is expected to increase from 1.7 million in 1990 to 6.3 million by 2050: a three-fold rise. In addition to the discomfort to the sufferer, osteoporosis will have a heavy impact on healthcare systems, due to the significant increase in morbidity, the high mortality risk and its subsequent severe effects on quality of life. Likewise, allergy affects 20% of the population, manifesting above all as asthma and allergic rhinitis. Actually, allergy is a complex disease of systemic nature and the most obvious demonstration of this is the development of different manifestations to a given allergen throughout the life of the same individual. [10]. Increased rates of musculoskeletal problems are reported in adults with allergic disease and muscle, joint, and bone problems must be considered also in children suffering from severe allergic disease [11].

### 2.1. Osteoimmunology

It is now accepted that there is an interconnection between immune cells and bone, and that several interactions occur in the bone microenvironment, such as the priming of naive T cells, the recruitment of effectors T cells, and their proliferation. Immune and bone cells derive from the same progenitors in the bone marrow and are sensitive targets of the same mediators regulating hematopoiesis, bone cells, and local immune responses. Furthermore macrophages, mast cells, B lymphocytes, natural killer cells etc. influence bone cells [12]. Cytokines provide instructional signals to bone marrow progenitors that differentiate into many subsets of innate effector cells of myeloid and lymphoid lineages, crucial components of different types of immune responses [13]. The fate of hematopoietic stem cells is driven by autocrine and paracrine mechanisms and bone tissue is involved in the process. Immune cells, osteoblasts, osteoclasts, and their progenitors seem to be able to communicate with each other, probably thanks to having many shared mediators. Macrophages, myeloid dendritic cells, and osteoclasts derive from the same myeloid precursor while osteoblasts regulate stem cell niches where all immune and blood cells originate [14]. Osteoclasts despite their heterogeneity in origin and environment are considered a single population of cells to be regarded as having immune capacities in addition to the well-known bone-resorbing role. This new vision of osteoclasts recognizes their leading role in the regulation of the bone immune status both in the steady state and during inflammatory processes [15]. The fact that osteoblasts can influence the myeloid lineage in the progression of preneoplastic and neoplastic transformations represents another strong connection between the immune system and bone [16].

### 2.2. Inflammation

**Figure 1:**
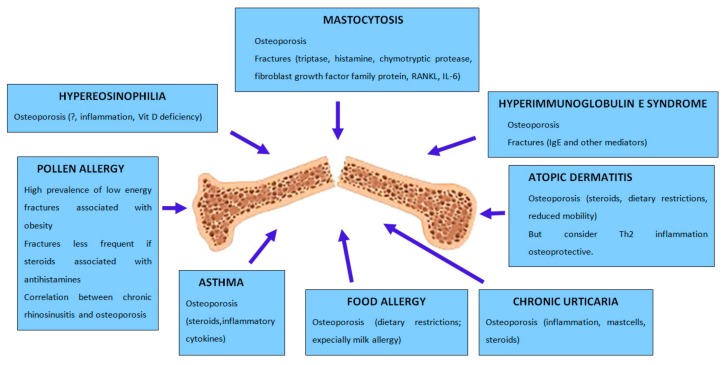
The figure summarizes the relationship between allergic diseases and skeletal health.

A connection exists between allergy and osteoporosis (Figure 1), two conditions that share common physiopathological and genetic risk factors, as supported by growing evidence from clinical and biological observations. A few researches suggest the existence of a link between allergy and bone loss, but there are several confounding factors and the evidence for a positive relationship is however weak. It is also true that, until now, little attention has been paid to the coexistence of osteoporosis and atopy [17].

What certainly connects the two diseases is the inflammatory process, its mechanisms, cells, and molecules involved and shared.

The immune system is designed to protect the organism, but sometimes inflammatory or allergic reactions may also lead to negative consequences. Asthma, eczema, and urticaria share these starting reactions involving the activation of T cells and several other types of cells and the production of histamine, cytokines and other mediators. Cytokines play a critical role in allergic intercellular interplay. They recruit and activate proinflammatory leukocytes contributing to disease pathology and to remodeling/pro-fibrotic events in chronic disease. In mild to moderate asthma, Th2 cytokines predominate, but in humans, inhibitors of IL-4 and IL-5 do not confirm the robust efficacy shown in animal models. It would seem evident that other important molecules are involved in driving pathology and confirm the complexity of allergic disease. The initiating allergic trigger can influence the Th2, Th1, and Th17 cytokines balance, highlighting the heterogeneity of the disease [18].

Several inflammatory cells, including eosinophils, mast cells, basophils, lymphocytes, dendritic cells and sometimes neutrophils participate to a complex interplay which generates the allergic inflammation. They produce reactive oxygen species, chemokines, cytokines, purines, lipids, all inflammatory mediators. Fibroblasts, epithelial cells, vascular cells, and airway smooth muscle cells are targets of allergic inflammation and also became relevant producers of inflammatory mediators.

Eosinophils and basophils are leading cells in the pathogenesis of airway allergic inflammation and are generated under the action of proinflammatory cytokines released after allergen challenge from the hematopoietic progenitors. Several transcription factors, particularly GATA3 and NF-kB, orchestrate allergic inflammatory response. On the other side, cytokines, hormones and growth factors cooperate to modulate bone metabolism, balancing osteoblast and osteoclast activity.

Interleukin-1, interleukin-6, and tumor necrosis factor alpha, proinflammatory cytokines, are main actors in bone metabolism and they also have a leading role in the pathogenesis of age [19] and estrogen deficiency related bone loss. Cytokines, hormones and growth factors finely cooperate to modulate bone metabolism balancing osteoblasts and osteoclasts activity. In most pathophysiological mechanisms intervenes the RANK/RANKL/OPG system, actively involved in the function and differentiation of osteoclasts [20,21]. RANKL play a leading role in osteclast differentiation and RANKL oversignaling produce excessive osteoclasts formation and bone resorption [22,23]. Mechanistically, when RANKL binds to RANK, its cell surface receptor, it triggers the activation of multiple downstream pathways: the nuclear factor-κB (NF-κB), mitogen-activated protein kinases (MAPKs), phosphatidylinositol 3-kinase/Akt (PI3K/Akt) [24], and phospholipase C gamma 2 (PLCγ2)-Ca^2+^-CREB signaling pathways, which leads to subsequent nuclear translocation of nuclear factor of activated T cells cytoplasmic 1 (NFATc1), a master transcription factor for osteoclastogenesis [25]. Osteoprotegerin (OPG), a decoy receptor for RANKL, secreted by osteoblasts and osteocytes is able to inhibit osteoclastic bone resorption by binding to RANKL and preventing its binding to RANK. Thus, the regulation of bone resorption, bone mass, and skeletal integrity are under control of the RANKL: OPG ratio that is modulated by a number of systemic factors. Recently, Yang et al. showed that OPG inhibits the survival and function of DC via the inhibition of RANK/RANKL signaling in asthmatic mice [26].

### 2.3. Osteopontin and Periostin

In this context two molecules seem interesting. One actor of bone remodelling, as activator of bone resorption process is osteopontin (OPN), an extracellular matrix protein and immune modulator with a wide range of functions. Postmenopausal women with osteoporotic vertebral fractures, low BMD, and increased levels of bone turnover markers show high levels of OPN. These data suggesting a significative role of OPN in physiopathology of postmenopausal osteoporosis are intriguing and necessitate further investigation [27]. OPN showed to be a pleiotropic cytokine with multiple roles in the regulation of allergic inflammation including cell migration, IgE regulation and development of airway fibrosis and angiogenesis, that functions locally and systemically [28]. Another interesting molecule is periostin (PO), physiologically involved in promoting tissue injury repair, found in the periostium of long bones and in many other organs and tissues including skin and lung [29]. In bone its highest expression is in periosteum and ostecytes controlled by several factors involved in bone remodelling such as mechanical stimuli, PTH, growth factors, and cytokines. Of particular interest is the finding that PO is highly expressed and plays an important role in chronic inflammatory diseases such as asthma, atopic dermatitis, eosinophilc chronic sinusitis/chronic rhinosinusitis with nasal polyp, and allergic conjunctivitis [30].

## 3. IL-33/IL-31 Axis

Recently was suggested that the IL-31/IL-33 axis could be involved in different conditions such as cancer, autoimmune diseases and allergies [31]. So, this is another interesting and new area to investigate among the complex overlapping mechanisms that connect allergies and osteoporosis (Table 1). Interleukin-31 (IL-31) is a proinflammatory cytokine, recently indicated as a biomarker of allergic and immunologic diseases, that regulates cell proliferation and play a role in tissue remodelling [31]. It is produced by CD4+ T lymphocytes (Th2 cells) and in lower amounts also by dendritic cells and mast cells. Its main targets are eosinophils and fibroblasts and IL-31 receptor is mainly expressed in non-hematopoietic tissue, skin and endothelium. It is associated with chronic skin inflammation and pruritus. An elevated IL-31 mRNA in the skin and increased serum levels of IL-31 are found in subjects with allergic contact dermatitis, atopic dermatitis, prurigo nodularis, chronic spontaneous urticaria, mastocytosis and primary cutaneous lymphoma and in some of these conditions IL-31 levels correlate with disease activity, as a diagnostic marker of severity in allergic diseases [32]. Several transcription factors and cytokines play a role in the development of osteoporosis and IL-31 is involved in their regulation. Janus-activated kinase/signal transducer and activator of transcription (JAK/STAT), phosphatidylinositol 3′-kinase/protein kinase (PI3K/AKT) and mitogen-activated protein kinase (MAPK) are the main signalling pathways through which IL-31 acts. These pathways are involved in bone remodelling and inflammation [24,32]. Increased levels of serum IL-31 are observed in postmenopausal women with a decrease of bone mineral density correlating with age, but not with the presence of fractures or osteoporosis degree [33].

Interleukin-33 (IL-33), a member of the interleukin-1 cytokine family, activates many tissue-resident immune cells expressing the heterodimeric receptor composed of suppression of tumorigenicity 2 (ST2) and IL-1 receptor accessory protein (IL-1RAcP) co-receptor. Identified as an alarmin and a nuclear factor controlling gene transcription, it warns immune system of barrier injury. The major targets of IL-33 appear to be mast cells, eosinophils, group 2 innate lymphoid cells (ILC2s), Th2 cells, regulatory T cells (Tregs), natural killer cells (NK), basophils, dendritic cells and alternatively activated macrophages, since ST2 is mainly expressed on these cells [34]. Large amounts of IL-5 and IL-13 are secreted by ILC2s responding to IL-33. Furthermore, the IL-33-ST2 activation of ILC2s contributes to anti-helminth responses and to the development of several allergic conditions such as atopic dermatitis, asthma, allergic rhinitis, and chronic rhino-sinusitis. Other non-allergic, inflammatory diseases, such as arthritis and periodontitis, seem to be involved in development and exacerbation the IL-33-ST2 mediated signalling. IL-33 show to be as potent as IL-3 and IL-5 in inducing eosinophil adhesion, eosinophil derived neurotoxin degranulation and increasing several cell surface markers expression [35]. This suggests a crucial role of IL-33 in modulating immune cells functioning in several pathologies such as asthma and lung diseases. IL-33 levels increase after cell death resulting in the induction of other cytokines including IL-31, however the full-length form of the molecule is cleaved by caspase 3 and 7 when cells undergo apoptosis [35]. IL-33 is known to protect from inflammatory bone loss inhibiting RANKL dependent osteoclast formation and inducing several anti-osteoclastogenic cytokines such as IL-10, IL-4, and IFN-g and granulocyte-macrophage colony-stimulating factor that inhibit osteoclast differentiation and to sum up it could be considered a bone protecting molecule [36].

A close relationship exists between these two interleukins and the IL-31/IL-33 axis has been indicated as a potential inflammatory pathway in chronic inflammatory diseases. The expression of one molecule is capable of inducing the production of the other, thus generating an amplification circuit of the inflammatory process and subsequent disease development. The role of IL-33/ST2 axis in Th2/IL-31 and Th17 immune response characterizing the development of allergic respiratory pathologies has been clarified while the relationship between the two cytokines in osteoporosis is still controversial [37]. Recently, there was reported an involvement of the IL-33/ST2 axis in the generation of Th17 cells and the production of IL-31 [37] and that the IL33/IL-31 axis is a link between osteoporosis and accelerated atherosclerosis in psoriatic arthritis [38]. A similar link can be hypothesized between allergic inflammation and osteoporosis.

### Autophagy

An increasing evidence suggest a key role of autophagy in several human diseases: it has emerged as a fundamental process in tissue and cellular homeostasis [39] and participates in the maintenance of bone marrow hematopoietic stem cell niche; it is critical for a well-balanced inflammatory response [40]. Autophagy seems to play a role in asthma pathogenesis where it may act as a potential contributor to airway inflammation, fibrotic airway remodelling [41], and airway hyper-responsiveness [42]. It can be either protective and detrimental as in the case of a prolonged exposure to allergen and inflammation, causing persistent or impaired autophagy. Recently, Lei et al. reported that autophagy might regulate IL-33 by inhibiting or activating NF-kB to control the uncontrolled inflammation of acute lung injury [43]. Interestingly, reactive oxygen species (ROS) are key mediators that contribute to oxidative damage and chronic airway inflammation in asthma and allergy. Environment/allergen exposure initiates the production of ROS in airway epithelial cells, thus serving as signaling molecule modulating autophagy and leading to the phenotypic changes of asthma listed above. In osteoimmunology, autophagy maintains cellular homeostasis during the differentiation of osteoclast and osteoblast, facilitates the function and survival of osteocyte, and regulates an immune response to limit inflammation and its resulting damage [44], and changes in this pathway seem to be related to osteoporosis [45]. The reduction of autophagy appears to increase oxidative stress causing bone loss. A wide range of cytokines regulate and are regulated by autophagy thus a better understanding of these processes may highlight the interplay and the crosstalk between diseases on the common ground of inflammation.

## 4. Vitamin D

Currently, vitamin D (VD), recognized as an immunomodulatory agent, represents a major link between allergy and bone. VD plays a leading role in calcium/phosphate balance and show effects on bone metabolism while demonstrating anti-inflammatory activity. It carries out important functions in immune system and may impact the course of immune-mediated pathologies. VD acts by skewing T lymphocytes to Th2 polarization and inhibits Th1 and Th17 lymphocyte activity and proliferation. [46]. The activation of Treg seem to be its main immunological activity. Some controversy exists surrounding the extra-skeletal role of VD, while the skeletal effects are better recognized.

Several observations suggest VD beneficial effects on pathologies related to hyperactivation of Th1 lymphocytes, such as rheumatoid arthritis, multiple sclerosis, psoriasis [47], diabetes type 1 and aspecific enteritis. In allergic diseases, where Th2 lymphocytes play a main role, VD has a controversial action. Several studies documented a beneficial effect of VD on the course of allergic diseases, although the underlying pathogenetic mechanisms have not yet been completely clarified [48].

The VD receptor is expressed in most body organs, including the heart, brain, prostate, gonads, skin, breast, gut, and in a multitude of cells such as osteoblasts, mononuclear cells, activated T and B cells, and beta cells. Epidemiology has often suggested a connection of VD deficiency with several diseases including allergies. Is reported the association of VD deficiency and asthma, and more specifically with increased airway inflammation, decreased lung function, increased exacerbations and poor prognosis. It may be that, by reducing inflammation in these patients, VD reduces the risk of respiratory infections and of exacerbations but does not reduce asthma severity [49,50]. The association between VD levels and allergies appeared weak in a recent cross-sectional study involving only allergic patients [51]. A possible role of VD in the eosinophil immune response is possible. These cells express the vitamin D receptor and VD prolong eosinophil survival and upregulate on their surface the C-X-C chemokine receptor type 4 (CXCR4). Moreover, VD reduces eosinophil necrosis and cytolytic release of peroxidase, reduces the production of immunoglobulin E (IgE) and increases expression of interleukin-10. Recently Filho et coll. showed that, in an unselected population not dominated by allergic patients, vitamin D deficiency is associated with a higher blood eosinophil count. The inverse correlation of vitamin D with the number of circulating basophils and neutrophils has been reported [52].

## 5. Histamine

Histamine is a molecule with a leading role in allergy. Bone cells express histamine receptors and regulation of bone metabolism may involve histamine. It has been shown that histamine promotes osteoclastogenesis through its receptors H1R, H2R and H4R [53,54]. Folwarczna et al. showed that the effects of histamine H1, H2, and H3 receptor antagonists in the skeletal system of rats depends on estrogen status and is differential. Loratadine a H1 receptor antagonist slightly favorably affect mechanical properties of compact bone [55]. Furthermore loratadine, often prescribed to allergic children, is able to slightly but significantly affect at high dose the development of the skeletal system in rapidly growing rats [56]. However, Aasarød et al. found increased levels of histamine in an animal model of gastric hypoacidity and hypergastrinemia associated with reduced bone quality and mechanical bone strength while the H1R antagonist cetirizine did not show to be beneficial on bone parameters [57,58,59,60].

## 6. Mast Cells

The best we know about mast cells (MCs) probably comes from allergic diseases, but they have relevant functions in maintaining tissue integrity. MCs have a strategic location in tissues exposed to external milieu in close proximity to nerves and blood vessels that allows a role of sentinels, sounding the alarm when they come into contact with harmful pathogens or stimuli. They constantly oversee their microenvironment for triggers such as infectious agents, hormones, toxins, alarmins, metabolic states, and promptly release inflammatory mediators, chemokines, cytokines, lipid products, allowing a coordinate response with resident tissue cells and rapidly recruiting immune effectors as well as the esocrine and exocrine system of the body. They play a main role in the IgE mediated hypersensitivity reactions of the gastrointestinal tract, the respiratory system, and the skin. In the last years, it has become clear that the physiopathological IgE response is individually modulated. The polyclonality of the immunoglobulin repertoire, the number of recognized epitopes, their affinity for antigens, and distance from each other influences the intensity of the release of mediators by MC. Skin mast cells express a specific and unique receptor the Mas related G protein coupled receptor X2 (MRGPRX2). This selective and highly expressed receptor promotes degranulation and mediator release in a different manner respect to IgE mediated activation. The MRGPRX2 cause a more transient effect, granules are relatively spherical and small and released immediately after activation. On the contrary, when activation is mediated by Fc receptor I, the secretion of larger and variously shaped granules probably due to their fusion, produce a delayed and more prolonged and intense response. In chronic urticaria and some pseudoallergic reactions, MRGPRX2 may be involved, but to date we lack information about the role of this receptor on mast cells in bone resorption [61]. Mast cells contribute to the allergic inflammation in a continuous crosstalk with all the components of the involved tissues through several soluble mediators. Among these a significative role is played by IL-31 and IL-33 [61], whose role has recently been investigated also in osteoporosis as described above [33,36,37]. When mast cells release repeatedly or continuously a wide range of molecules, metabolic changes may intervene, among these osteoporosis and bone remodelling. This is an indisputable fact in the case of proliferative diseases such as mastocitosis, but remains to be considered also in hypersensitivity diseases. Bone formation and turnover is affected by mast cells products such as histamine tryptase and others that influence the balance between osteoclasts and osteoblasts/bone degrading and bone formation. Mastocitosis is associated with high frequency to inflammatory joint disease [62] as a possible consequence of a dysregulation of the mast cell/regulatory lymphocytes interaction in the immune response, and in both conditions, we observe the alteration of bone mineralization. Bone metabolism alterations in asthma are mostly ascribed to steroid treatment but several observations suggest that this is not the only responsible factor. To date, however, we lack enough information concerning a specific and decisive role of mast cells in osteoporosis in allergic diseases. Mast cells are too abundant in mastocitosis and too active in allergy, and this suggests that the action on bone may be similar. Furthermore, MCs express the vitamin D receptor able to inhibit secretion of mediators induced by IgE when joined to its ligand [63] and this is another connection between these cells and bone metabolism.

## 7. Pollen-Allergy

In 2006, Ferencz et al. investigated bone mass changes and fractures in pollen-allergic postmenopausal women and found a high prevalence of low energy fractures associated with obesity. Fractures were less frequent in subjects under combined treatment with inhaled steroid and antihistamine respect to women only treated with inhaled steroid. Authors speculated that the H1 histamine receptor antagonists compensate the negative action on bone of both corticosteroid therapy and pollen allergy [64]. Recently, Choi and Kong [65] in their case-control study showed an association between chronic rhinosinusitis and osteoporosis, but they could not confirm a physiopathological mechanism linking the two diseases nor correlating them with drug intake. They only speculated the occurrence of osteoporosis as with other chronic inflammatory conditions. Contrasting results from the study by Gelardi et al. that assessing bone metabolism in chronic rhinosinusitis associated with nasal poliposis and long-term therapy with corticosteroids, showed that the likelihood of osteoporosis or osteopenia between affected and healthy subjects was superimposable with no statistically significant parameter predicting alterations of bone metabolism [66].

## 8. Asthma

The association of osteoporosis with asthma is known, but in the past, most studies mainly focused on the bone adverse effects of prolonged corticosteroid treatments [67,68]. Recent studies have emphasized the role of the systemic inflammatory response to the chronic lung inflammatory disease in the development of osteoporosis, as the pivotal role of inflammatory mediators in this condition is well known. Osteoporosis is more common in subjects affected by chronic obstructive pulmonary disease (COPD) than in patients with asthma, even not receiving steroidal therapy. Thus, demonstrating the role of inflammatory cytokines such as tumor necrosis factor alpha inducing osteoclastic bone resorption. A more severe degree of osteoporosis in COPD with respect to asthma is the result of a higher systemic inflammation, and consequently of higher circulating inflammatory cytokines observed in COPD. Osteoporosis tends to be more prevalent in subjects with asthma-COPD overlap than in those with asthma and the effects on bone of inhaled corticosteroids are less significant than systemic inflammation [69]. A multitude of effector cells such as neutrophils, eosinophils, basophils, mast cells, macrophages, monocytes, T, B, and NK cells, participate in inflammation allergy and asthma, leading to a cascade of events and the release of several mediators activating diverse biological effects and immune responses. Among these, eicosanoids, the major precursors in cyclooxygenase and lipooxygenase pathways that play a key role [70]. The Cysteinyl leukotrienes (CysLTs) are a family of potent inflammatory lipid mediators derived from arachidonic acid through the 5-LO pathway. The cysteinyl leukotriene receptor 1 (CysLTR1) is a G-protein coupled receptor (GPCRs) and its interaction with CysLTs plays a central role in the pathophysiology of asthma and other inflammatory diseases. Montelukast is a CysLTR1 antagonist clinically used for the treatment of asthma. Kang et coll. showed that montelukast potently prevents RANKL-induced osteoclasts formation and bone loss in vivo [71].

## 9. Atopic Dermatitis

Osteoporosis and fracture risk are recognized long-term sequalae of atopic dermatitis (AD), a common inflammatory skin disease [72,73]. The association between bone weakness and AD may have several potential reasons. AD is associated with type 2 inflammation that may result in protection rather than increased risk of fracture. Indeed, Th2 (and Th1) cytokines inhibit osteoclastogenesis with an osteoprotective action. They enhance the anabolic effects of paratormone and decrease the receptor activator of nuclear factor-kb ligand/osteoprotegerin, inhibiting bone resorption [74]. AD may influence the achievement of a normal peak bone mass due to dietary restrictions with suboptimal calcium and vitamin D intake at a critical period of bone mineralization, mostly when the disease arises early in childhood and associated with food allergies. Furthermore, in severe AD, it may be hard to mobilize, and the reduced physical activity contribute to osteopenia. Last but not least, glucocorticoids are recognized major risk factor for osteoporosis and fractures [8], a commonly used therapeutic weapon in AD. Even with potent topical steroids, there is little or no evidence of increased fracture risk and there are contrasting data on the possibility that inhaled corticosteroid to treat asthma associated with severe AD may increase the risk of fracture. Further, it remains important to consider the significant difference in vitamin D levels detected between patient with or without atopic dermatitis [51]. Finally we highlight that periostin correlate with disease severity and chronicity in AD [75].

## 10. Chronic Urticaria

Epidemiological evidence is reported on the association between chronic urticaria (CU) and osteoporosis [76]. CU as a low grade inflammatory condition [77,78,79] is a potential risk factor for osteoporosis, to which the female predisposition and the exposure to systemic corticosteroids is added. We do not have physiopathological certainties on the high prevalence of osteoporosis in CU patients, but only speculations about the possible roles of chronic inflammation and mast cell abnormalities connecting these two pathologies. Accelerated bone absorption is associated to mast cell excess while their deficiency with low remodelling states. Mastocytes in osteopenia and osteoporosis secrete cytokines active on bone balance: bone turnover shift towards decreased bone formation and increased bone resorption. We have limited knowledge on the possible beneficial effect of mast cells inhibition in humans to improve skeletal health.

## 11. Milk-Allergy

Many factors may disturb maintenance of healthy bones including inflammatory states and poor nutrition. A significantly positive association was demonstrated between skeletal health and dairy food intake [80] and young adults with IgE mediated cow’s milk allergy (IgE-CMA) show significantly lower bone mineral density (BMD) than gender and age matched controls [81]. The best source of bioavailable calcium is milk that also contain proteins just as important to bone health, such as milk-derived ribonuclease that promotes angiogenesis within bone tissue and lactoferrin which is capable of stimulating osteoblast differentiation and reduce bone resorption [82]. Abnormal BMD scores are found in IgE-CMA patients, but analysis of results suggest that additional factors may play a role in these scores. Asthma and weight in the context of a low calcium intake are independent risk factors for reduced BMD and in the IgE-CMA population is frequent to experience other manifestations of the atopic march. A study found that infants with cow milk allergy have lower levels of vitamin D [83,84].

## 12. Mastocytosis

In mastocytosis, a disease with abnormal growth and the accumulation of mast cells and symptoms mostly related to released mediator effects, reduced bone mass has been described due to increased bone resorption and normal or decreased bone formation [64]. In 70% of patients with systemic mastocytosis, bone involvement has been observed with a high prevalence of osteoporotic fractures [85,86,87,88]. Osteoporosis is due to neoplastic infiltration of mast cells and the effects of the mediators that they release. Histamine acting through four different receptors is responsible for the most relevant clinical actions: pruritus, vasodilatation, vasopermeability, bronchial and gastrointestinal smooth muscle contraction and gastric acid secretion. Bone remodelling is the result of the effect of tryptase together with chymotryptic proteases, histamine, fibroblast growth factor family proteins, RANKL and IL-6 [61].

## 13. Hyperimmunoglobulin E syndrome

A rare form of immunodeficiency characterized by increased serum levels of IgE associated with a tendency to fractures and osteopenia is the hyperimmunoglobulin E syndrome (HIES). Scheuerman and Sowerwine documented a bone density decrease in autosomal dominant HIES [89,90]. In this condition, there is an imbalance of cytokine secreting lymphocyte subpopulation and some of these cytokines play a role in bone remodelling. Mononuclear cells from these patients show an increased bone resorption activity similar to that of postmenopausal women suggesting that in HIES IgE related mechanism could have a leading role in driving to bone loss [91]. Furthermore, osteoclasts from HIES subjects with STAT3 mutation have higher bone resorption activity than those from healthy subjects [92]. More than 50% of affected people have recurrent pathological fractures as a result of minor injuries, typically long bones and ribs, and less frequently the spine as a consequence of the multisystemic involvement of the disease and a reduced bone mineral density do not appear to be a predisposing factor for fractures in these patients [93].

## 14. Conclusions

**Figure 2:**
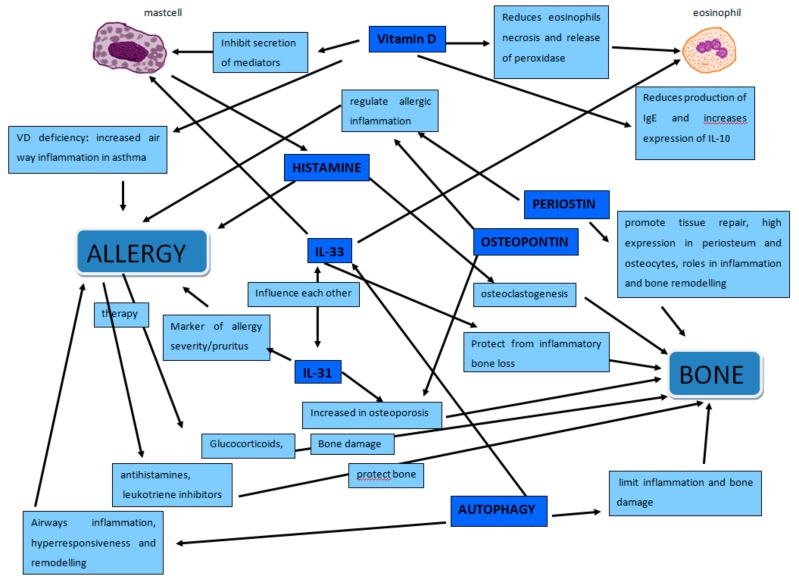
The figure shows the complex network of relationships between allergy and bone. Mast cells that play a leading role in allergy but also in the regulation of bone metabolism through histamine, tryptase and other molecules. Osteopontin that regulate allergic inflammation and increase in osteoporosis. Periostin that regulate allergic inflammation and intervene in bone remodelling. IL-31 marker of allergic inflammation that increases in osteoporosis. IL-33 that influences both mast cells and eosinophils, modulate allergic inflammation and protect from inflammatory bone loss. Vitamin D, regulator of calcium/phosphate balance, recognized immunomodulatory agent, that inhibit secretion of mediators by mast cells, reduces eosinophils necrosis, release of peroxidase, production of IgE and increases expression of IL-10. Autophagy that regulate IL-33, in osteoimmunology limit inflammation and bone damage while in asthma contribute to airways inflammation, hyperresponsiveness and remodelling.

In this review, we have summarized and discussed the many factors that link allergies and bone metabolism to give an updated report on this topic (Figure 2). We tried to look through epidemiological, clinical, and laboratory data to highlight these connections. Molecules, cells, diseases, and drugs constitute the different perspectives we took to look at this issue.

If the clinical correlation between proliferative pathologies such as mastocytosis or hyper-IgE syndrome and skeletal pathology is historically known, the most recent epidemiological data confirm a link between osteoporosis and pollen-allergy, asthma, atopic dermatitis, urticaria, milk allergy. Equally certain is the link between bone pathology and therapies adopted in the treatment of diseases with allergic etiology. No longer are steroids alone responsible, but antihistamines, pump inhibitors, antileukotrienics. On the other hand, it is more difficult to go into an interconnected pathogenetic hypothesis of the two diseases. An inflammatory picture is the fundamental pathogenetic moment for both, and the cellular and humoral protagonists are mostly the same, however it is easy to imagine how different balances and modulations in both cases can be difficult to read and interpret. A leading mediator of allergy, histamine, has been shown to have a role in the determinism of bone pathology, and in the same way vitamin D which has a leading role in bone metabolism affects the modulation of allergic diseases. Mast cells, leading actors of allergic inflammation, produce molecules that affect bone formation and turnover. The signal pathway of NF-kB plays a major role both in allergic inflammation and in regulating bone resorption and it is suggested as a potential therapeutic target [94]. The two Th2 cytokines IL-31 and IL-33 demonstrated to be relevant in the pathogenesis of both allergy and osteoporosis as well as autophagy and these too have been suggested as potential therapeutic target [33,36,37,42]. The role of so many actors may vary and their final effect depends on multiple factors, including their mutual interaction and ultimately the combined action in the different phases of inflammation and/or bone remodelling.

While confirming that allergy and bone metabolism have common mediators and pathways, the available data are heterogeneous and sometimes conflicting. A great certainty is that it is a matter of extreme complexity still in an embryonic phase of study that requires further targeted and in-depth studies. The aim of the work was to summarize the current knowledge on the subject to make clinicians aware of the preventive and therapeutic implications and to stimulate researchers on new intriguing hypotheses of study.

## Figures and Tables

**Table 1:** Bone, allergic diseases, and the IL-33/IL-31 axis.

	IL-31	IL-33
**Bone**	Involvement in postmenopausal osteoporosis	Protective
**Atopic dermatitis**	Involvement in impairment of skin barrier function	Modulate eosinophil function. IL-33 inhibition: reduced IgE serum IgE levels, and mast cells and eosinophils infiltration
**Asthma and allergic rhinitis**	Asthma exacerbation	Inflammation and fibrotic damage. IL-33 inhibition: asthma improvement
**Allergic contact dermatitis**	Pruritus	Early warning system of skin damage. Inhibit contact hypersensitivity and induce Treg; IL-33 blockade worsens contact hypersensitivity.
**Chronic spontaneous urticaria**	Pruritus	
**Food allergy**		Th2 response promotion. IL-33 inhibition: FA improvement
